# Non-Human Primate iPSC Generation, Cultivation, and Cardiac Differentiation under Chemically Defined Conditions

**DOI:** 10.3390/cells9061349

**Published:** 2020-05-29

**Authors:** Michael Stauske, Ignacio Rodriguez Polo, Wadim Haas, Debbra Yasemin Knorr, Thomas Borchert, Katrin Streckfuss-Bömeke, Ralf Dressel, Iris Bartels, Malte Tiburcy, Wolfram-Hubertus Zimmermann, Rüdiger Behr

**Affiliations:** 1 Research Platform Degenerative Diseases, German Primate Center–Leibniz Institute for Primate Research, Kellnerweg 4, 37077 Göttingen, Germany; IRodriguezPolo@dpz.eu (I.R.P.); wadim.haas@mail.de (W.H.); debbrayasemin.knorr@uni-goettingen.de (D.Y.K.); 2 DZHK (German Centre for Cardiovascular Research), Partner Site Göttingen, 37077 Göttingen, Germany; thomasborchert_tb@hotmail.com (T.B.); katrin.streckfuss@med.uni-goettingen.de (K.S.-B.); rdresse@gwdg.de (R.D.); m.tiburcy@med.uni-goettingen.de (M.T.); w.zimmermann@med.uni-goettingen.de (W.-H.Z.); 3 Department of Cardiology and Pneumology, University Medical Center Göttingen, Robert-Koch-Str. 40, 37075 Göttingen, Germany; 4 Institute of Cellular and Molecular Immunology, University Medical Center Göttingen, Robert-Koch-Str. 40, 37075 Göttingen, Germany; 5 Institute of Human Genetics, University Medical Center Göttingen, Robert-Koch-Str. 40, 37075 Göttingen, Germany; iris.Bartels@posteo.de; 6 Institute of Pharmacology and Toxicology, University Medical Center Göttingen, Robert-Koch-Str. 40, 37075 Göttingen, Germany

**Keywords:** induced pluripotent stem cell, cellular reprogramming, cardiac differentiation, Wnt signaling, translation, rhesus monkey, non-human primate

## Abstract

Non-human primates (NHP) are important surrogate models for late preclinical development of advanced therapy medicinal products (ATMPs), including induced pluripotent stem cell (iPSC)-based therapies, which are also under development for heart failure repair. For effective heart repair by remuscularization, large numbers of cardiomyocytes are required, which can be obtained by efficient differentiation of iPSCs. However, NHP-iPSC generation and long-term culture in an undifferentiated state under feeder cell-free conditions turned out to be problematic. Here we describe the reproducible development of rhesus macaque (*Macaca mulatta*) iPSC lines. Postnatal rhesus skin fibroblasts were reprogrammed under chemically defined conditions using non-integrating vectors. The robustness of the protocol was confirmed using another NHP species, the olive baboon (*Papio anubis*). Feeder-free maintenance of NHP-iPSCs was essentially dependent on concurrent Wnt-activation by GSK-inhibition (Gi) and Wnt-inhibition (Wi). Generated NHP-iPSCs were successfully differentiated into cardiomyocytes using a combined growth factor/GiWi protocol. The capacity of the iPSC-derived cardiomyocytes to self-organize into contractile engineered heart muscle (EHM) was demonstrated. Collectively, this study establishes a reproducible protocol for the robust generation and culture of NHP-iPSCs, which are useful for preclinical testing of strategies for cell replacement therapies in NHP.

## 1. Introduction

**Figure 1:**
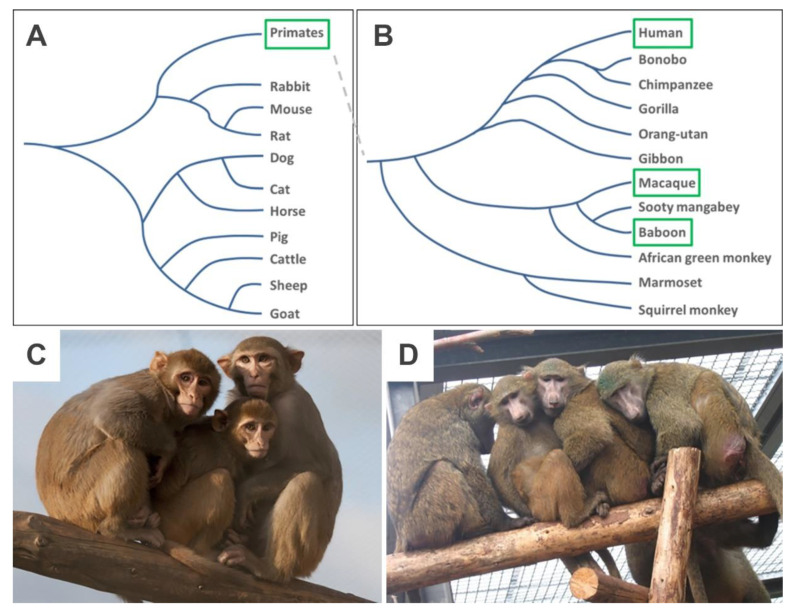
Non-human primates in basic and translational research. (**A**) Phylogenetic tree of mammals used as model organisms in research (after [5]), (**B**) phylogenetic tree of selected primates (after [3]), (**C**) rhesus macaques, (**D**) olive baboons. Photographic images by German Primate Center (DPZ).

The value of non-human primates (NHP) as animal models in translational research is increasingly recognized [1]. NHP offer advantages compared to other species because of their close phylogenetic relationship to humans [2,3,4] (Figure 1).

This is reflected by similar anatomy, physiology, and, most importantly, a similar genotype [4,6,7,8,9]. Collectively, these characteristics make NHP excellent surrogates for the preclinical testing of human medicines. The relevance of NHP models is further exemplified by their widespread use in safety and efficacy studies [10,11,12,13,14,15]. NHP models have been introduced for the late preclinical testing of pluripotent stem cell (PSC)-based advanced therapy medicinal product (ATMP) candidates in the fields of cardiology and neurodegenerative diseases [16,17,18,19,20,21,22,23,24]. These studies include the testing of human xenografts [17,19,20,24] and NHP allo- as well as autografts [16,21,23,24]. Moreover, a variety of protocols for the generation and cultivation of NHP induced pluripotent stem cells (iPSCs) have been published; all of them, to our knowledge, require feeder cells and/or xenogenic serum [16,21,23,25,26,27,28,29]. Moreover, several iPSC lines were generated using integrating retroviral vectors [23,29,30]. It is generally recognized that the reprogramming of NHP cells is less efficient in comparison to the well-established human reprogramming techniques [31,32], although this has not been systematically quantified so far. For instance, it has been demonstrated that marmoset monkey iPSC generation required five or even six reprogramming factors [33,34,35], and marmoset cell reprogramming with the four Yamanaka factors was successful only after around 100 days [36]. In agreement with these observations, also low efficiency of macaque iPSC line generation has been recognized [23].

In this study, we confirmed lower reprogramming efficiency of rhesus macaque fibroblasts compared to human fibroblasts. Yet, we established a versatile and reproducible protocol for vector integration- and feeder-free NHP-iPSC generation from rhesus macaques (*Macaca mulatta*) and baboon (*Papio anubis*). Furthermore, we critically refined culture conditions to enable long-term maintenance of rhesus and baboon PSCs in an undifferentiated state. These culture conditions were also successfully applied to human cells. Finally, we tested several protocols for the directed differentiation of NHP-iPSCs to cardiomyocytes and identified a combined growth factor and Wnt-modulation protocol to be most efficient.

## 2. Materials and Methods

### 2.1. NHP Species and Ethics Statement

Two NHP species with large body size, the rhesus macaque (*Macaca mulatta*; 4–14 kg body weight) and the olive baboon (*Papio anubis*; 10–37 kg body weight), were included. Both species show a close phylogenetic relationship to humans (Figure 1) and are widely used in cardiovascular research [17,21,37,38,39]. The permission and ethical approval to take skin biopsies from the macaques were obtained under the license number 42502-04-16/2370 from the Niedersächsisches Landesamt für Verbraucherschutz und Lebensmittelsicherheit (LAVES). The baboon skin sample was made available to the Platform Degenerative Diseases of the German Primate Center (DPZ) during necropsy of the animal in an unrelated study by veterinarian pathologists. Care of animals was in accordance with all legal and institutional guidelines.

### 2.2. Isolation and Cultivation of NHP and Human Skin Fibroblasts

Fibroblasts from five rhesus macaques (one newborn and four adult individuals including males and females) and one adult female baboon were isolated from outgrowths from small skin samples and cultured in fibroblast culture medium (89 mL DMEM, 10 mL fetal bovine serum (FBS), 1 mL 100× non-essential amino acids (NEAA), 0.1 mM 2-mercaptoethanol; all Thermo Fisher, Waltham, MA, USA) supplemented with 10 ng/mL bFGF (PeproTech, Hamburg, Germany) and penicillin-streptomycin (Thermo Fisher). Cell outgrowths were dissociated and split using 0.25% trypsin/EDTA (Thermo Fisher) and propagated in fibroblast medium containing bFGF, but without further antibiotic treatment. Human skin fibroblasts were purchased from Lonza (CC-2511, lot 0000545147 [male], lot 0000490824 [female]) and cultured under the same conditions.

### 2.3. Reprogramming NHP and Human Skin Fibroblasts

Fibroblasts were nucleofected with the three episomal vectors pCXLE-hOCT3/4-shp53-F (#27077), pCXLE-hSK (#27078) and pCXLE-hUL (#27080) [40] using the 4D-Nucleofector Core Unit and the P2 Primary Cell Solution (Lonza, Basel, Switzerland). The cells were transfected with 2 μg of each plasmid (per 6E05 cells) and plated onto 6-well plates (3E05 cells per well; Greiner Bio-One, Frickenhausen, Germany) in fibroblast culture medium supplemented with 10 ng/mL bFGF, 5 μM pro-survival compound (aka ROCKi; Merck, Darmstadt, Germany) and penicillin-streptomycin. The next day, the medium was replaced by fresh fibroblast medium containing 10 ng/mL bFGF and 0.5 mM sodium butyrate (Sigma-Aldrich, Taufkirchen, Germany). The medium was changed daily. On day 7 after transfection, the cells were spilt onto geltrex (0.16 mg/mL; Thermo Fisher) coated 6-well plates. At day 8, fibroblasts culture medium was replaced by Essential 8 medium (Thermo Fisher) supplemented with 0.5 mM sodium butyrate until day 11. From day 12 on, the transfected cells were cultured in Essential 8 medium only. Appearing iPSC colonies were picked manually and transferred into geltrex-coated 12-well plates (Greiner Bio-One). For continuous cultivation and expansion, the medium was replaced by a newly developed medium named universal primate pluripotent stem cell (UPPS) medium (see below).

For quantification of the reprogramming efficiency, the skin fibroblasts of three rhesus macaques and two humans were transfected and cultured as described above. The nucleofected fibroblasts were split into three wells of a 6-well plate at day 7. The appearing colonies were stained for alkaline phosphatase and counted at day 20, 30, and 40 after nucleofection (colonies counted in one well at each time point). Alkaline phosphatase activity was detected using an alkaline phosphatase staining kit (Sigma-Aldrich).

### 2.4. Cultivation of Human and NHP-PSCs

Human and NHP-PSCs were maintained in UPPS medium, which is based on StemMACS iPS Brew XF (Miltenyi Biotec, Bergisch Gladbach, Germany) supplemented with 1 μM inhibitor of Wnt response-1 (IWR-1, Sigma-Aldrich) and 0.5 μM CHIR99021 (Merck). The stem cells were cultured on geltrex coated (0.16 mg/mL) culture dishes at 37 °C and 5% CO_2_. The cells were split every 3–4 days onto new culture dishes using Versene solution (Thermo Fisher). For cell passaging, the UPPS medium was supplemented with 5 μM pro-survival compound (ROCKi) for one day.

### 2.5. In Vivo and In Vitro Differentiation

For in vivo differentiation experiments (teratoma formation), human and NHP-iPSC colonies were treated with collagenase type IV (200 U/mL; Worthington) for 10 min at 37 °C, washed and dissected into small cell clusters using a cell scraper (Sarstedt, Nümbrecht, Germany) followed by pipetting smoothly up and down. The cell clusters were centrifuged and resuspended together with irradiated mouse embryonic fibroblasts (to enhance cell survival and teratoma formation) in 140 μL UPPS medium supplemented with 5 μM pro-survival compound (ROCKi) and 120 μL geltrex (0.16 mg/mL). This suspension was then injected subcutaneously into SCID/beige (C.B-17/IcrHsd-scid-bg) mice. The earliest teratoma formation was detected one-month post-injection. The teratomas were fixed with Bouin’s fixative for 24 h and embedded into paraffin. Histological sections were stained with hematoxylin and eosin (Merck) or with antibodies against SOX9 and β-tubulin III (Table 1).

NHP-iPSCs were also differentiated in vitro using the embryoid body (EB) formation method. Briefly, after treatment with collagenase type IV, cell clusters were cultured in suspension in UPPS medium for 24 h giving them time to form EBs. The next day, the medium was replaced by differentiation medium (79 mL IMDM (Thermo Fisher), 20 mL FBS, 1 mL 100× NEAA, 450 μM 1-thioglycerol (Sigma-Aldrich)). At day 8, the EBs were plated onto 0.1% gelatin-coated coverslips (ø 25 mm; Fisher Scientific, Schwerte, Germany) and fixed for immunostaining at day 25.

### 2.6. Cardiac Differentiation

Different protocols for cardiac differentiation established for human PSCs were tested for our rhesus and baboon iPSCs. Three tested basic approaches (Table 2) were based on the small molecule CHIR99021 with and without the growth factors, bone morphogenetic protein 4 (BMP4) and activin A.

**Figure 2:**
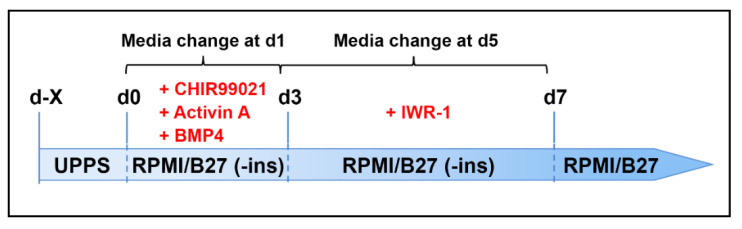
Scheme of the cardiac differentiation protocol (hybrid method).

In contrast to human PSCs, the growth factor-free cardiac differentiation protocols (see Appendix A) did not work for NHP-PSCs in our hands. However, the hybrid differentiation method [41], including CHIR99021, activin A and BMP4, was successful (Figure 2).

When reaching a confluency of about 80–90%, the UPPS medium was replaced by mesodermal induction medium (RPMI 1640, B27 supplement without insulin, 1 mM sodium pyruvate (Thermo Fisher), 200 μM L-ascorbic acid 2-phosphate, 1 μM CHIR99021, 9 ng/mL activin A (Miltenyi Biotec, premium grade), 5 ng/mL BMP4 (Miltenyi Biotec, premium grade). Medium was changed after 24 h. At day 3, medium was replaced by cardiac induction medium (RPMI 1640, B27 supplement without insulin, 1 mM sodium pyruvate, 200 μM L-ascorbic acid 2-phosphate, 5 μM IWR-1). Medium was changed at day 5. At day 7, the medium was replaced by cardiomyocyte culture medium (RPMI 1640, B27 with insulin, 200 μM L-ascorbic acid 2-phosphate). The cultures were monitored daily for contractile activity. To increase the yield of pure cardiomyocytes, the cells were metabolically selected with lactate [42]. Briefly, the cells were plated onto geltrex coated 6-well plates around day 20 after starting differentiation. The cells were then cultured for 4 or 5 days in cardiac selection medium containing lactate instead of glucose (RPMI 1640 without glucose (Thermo Fisher), 4 mM lactate/HEPES solution, 0.2 mg/mL L-ascorbic acid 2-phosphate, 0.5 mg/mL recombinant human albumin). After cardiomyocyte selection, the medium was changed back to cardiomyocyte culture medium.

### 2.7. Immunostaining

Cells were fixed with 4% (*w*/*v*) paraformaldehyde (Merck) in DPBS (Thermo Fisher) for 20 min at room temperature and subsequently washed with DPBS. After blocking with 1% bovine serum albumin (BSA; Thermo Fisher), the cells were incubated with primary antibodies (Table 1) at 4 °C overnight. For staining intracellular proteins, the cells were additionally treated with 0.1% triton X-100 (Sigma-Aldrich). After washing with DPBS, the cells were incubated with secondary antibodies (Table 1) for 1 h at 37 °C. Cell nuclei were stained with 1 μg/mL DAPI (Sigma-Aldrich). The cells were finally mounted with Fluoromount–G (Thermo Fisher) and analyzed with a fluorescence microscope (Zeiss, Oberkochen, Germany). Immunohistochemical staining of the teratomas was performed as described recently [43].

### 2.8. Flow Cytometry

At day 12 after initiation of differentiation, human and NHP-iPSC-derived cardiomyocytes were dissociated with 0.25% trypsin/EDTA, flushed through a 40 μM cell strainer (Fisher Scientific, Schwerte, Germany) and single cells were fixed with 4% (*w*/*v*) paraformaldehyde for 10 min. After a blocking step with 1% BSA at 4 °C, the cells were incubated with FITC-conjugated cTNT antibody solution (Table 1) diluted in 0.1% triton X-100/BSA at 4 °C overnight. The next day, cells were washed and resuspended in 100 μL flow cytometry buffer (0.5% BSA, 2 mM EDTA (Carl Roth, Karlsruhe, Germany)). Unstained cells from each cell suspension in the presence of non-specific antibody of the same Ig class were used as negative control. Flow cytometric analyses (10,000 cells per sample) were performed with SH800S Cell Sorter using a 130 μm sorting chip (Sony Biotechnology, Weybridge, UK) according to the manufacturer’s instructions.

### 2.9. DNA and RNA Isolation and Polymerase Chain Reaction

Genomic DNA from snap-frozen cell pellet samples (between passage 15 and 25) was isolated using the DNeasy Blood & Tissue Kit (Qiagen, Hilden, Germany) according to the manufacturer’s instructions. To confirm the loss of episomal plasmids in the generated iPSCs, we performed a standard polymerase chain reaction (PCR) using plasmid-specific primer pairs (Table 3), which are complementary to four different regions present in all three plasmids. One PCR reaction (50 μL) was performed using 50 ng of genomic DNA, following the manufacturer’s instructions using Taq DNA Polymerase with Standard Taq Buffer (New England Biolabs, Frankfurt, Germany).

For gene expression analyses, total RNA from snap-frozen cell pellets was isolated using a NucleoSpin RNA Plus isolation kit (Macherey-Nagel, Düren, Germany), treated with RNase-free DNase (Qiagen, Hilden, Germany) and subsequently transcribed into cDNA with Omniscript RT kit (Qiagen, Hilden, Germany) according to the manufacturer’s instructions. The cDNA was then amplified using Taq DNA polymerase with Standard Taq Buffer (New England Biolabs, Frankfurt, Germany). All oligonucleotides (Sigma-Aldrich) are listed in Table 3.

### 2.10. Karyotyping

After long-term cultivation (>30 passages), the generated iPSC lines were karyotyped as described previously [44].

### 2.11. Microelectrode Array (MEA) Measurements

The iPSC-derived cardiomyocytes were dissociated with 0.25% trypsin/EDTA and plated onto geltrex coated 6-well MEAs (Multichannel Systems, Reutlingen, Germany) at a density of 25,000 cells per MEA well. After 4–7 days of recovering, field potentials of the cardiomyocytes were measured using the MEA2100-2 × 60-System at 38 °C (physiological body temperature of macaques). After basal field potential measurements, the cells were treated with the non-selective β-adrenoreceptor agonist isoprenaline (100 nM; Sigma-Aldrich) followed by propranolol (2 µM; Sigma-Aldrich) after 5 min. The effect on the beating frequency was detected by continuously measuring the field potentials of the cardiomyocytes. Data were acquired and analyzed using MC_Rack software (Multichannel Systems, Reutlingen, Germany).

### 2.12. Engineered Heart Muscle (EHM) Generation

EHMs from human and NHP-iPSC-derived cardiomyocytes were generated to determine contractility as previously described [41]. In brief, metabolically selected cardiomyocytes were mixed with human foreskin fibroblast (HFFs, American Type Culture Collection; 70:30). Cell solution was reconstituted in pH-neutralized medical-grade bovine collagen (0.4 mg per EHM; Collagen Solutions, Glasgow, UK) and concentrated serum-free medium (2× RPMI, 8% B27 supplement without insulin, penicillin [200 U/mL], streptomycin [200 μg/mL]). The EHMs were cultivated for 3 days in IMDM with 4% B27 supplement without insulin, 1% NEAA, 2 mM glutamine, 300 μM L-ascorbic acid, 100 ng/mL IGF1, 10 ng/mL FGF-2, 5 ng/mL VEGF165, 5 ng/mLTGF-β1 (all growth factors from PeproTech, Hamburg, Germany), penicillin (100 U/mL), and streptomycin (100 μg/mL). After 3 days, the EHMs were transferred to flexible holders, and 4 weeks later, EHM analysis was performed as described.

### 2.13. Statistics

Statistical analysis was performed using GraphPad Prism 8 software (San Diego, CA, USA). Graphs are represented as mean with standard deviation or fold change.

## 3. Results

### 3.1. NHP and Human Fibroblast Reprogramming

**Figure 3:**
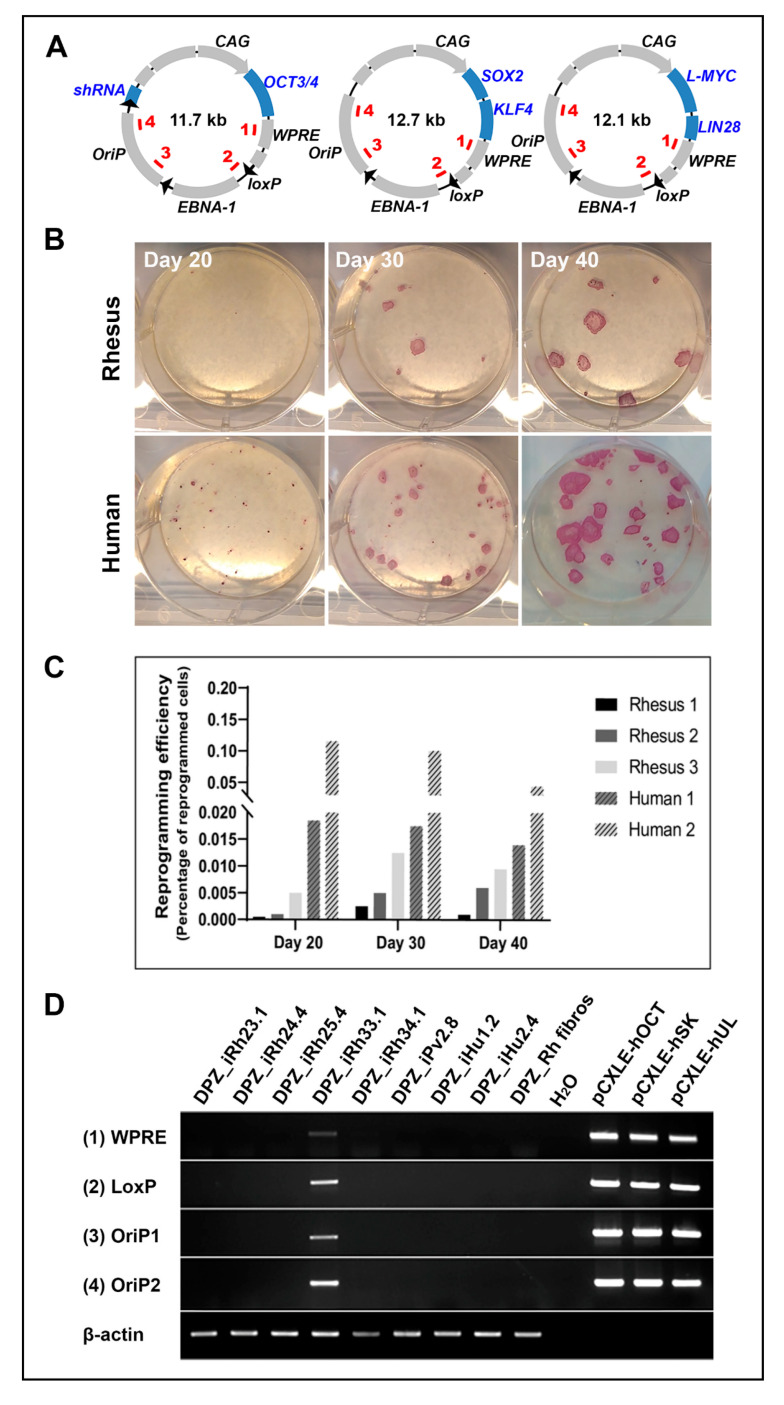
Comparison of reprogramming efficiencies between rhesus and human fibroblasts using episomal plasmids. (**A**) Reprogramming vectors used in this study (modified after [40]). Primer-binding sites (1–4) for plasmid detection are depicted within the plasmid DNA. (**B**) To compare reprogramming efficiencies, skin fibroblasts from three different rhesus macaques and two human donors were nucleofected and colonies were stained for alkaline phosphatase and counted at day 20, 30, and 40 after nucleofection. Representative staining, shown for Human 1 and Rhesus 1. (**C**) Reprogramming efficiency shown as the percentage of reprogrammed cells. The colony count revealed lower reprogramming efficiencies of the rhesus cells compared to the human cells. (**D**) The absence of plasmid DNA in the generated induced pluripotent stem cell (iPSC) lines was analyzed by PCR on gDNA using primers specific for four different regions of the plasmid DNA. Only the rhesus iPSC line DPZ_iRH33.1 shows integration of plasmid DNA. Rhesus fibroblasts and water were used as negative, the plasmids as positive controls.

We aimed at generating integration-free NHP- and human iPSCs under feeder- and serum-free conditions using an easy and cost-effective reprogramming method. Therefore, we used established episomal vectors [40] (Figure 3A). To quantify and compare reprogramming efficiencies, we investigated reprogramming in primary fibroblast cultures obtained from three adult rhesus macaques and two human adults. Primary colonies were counted after staining for alkaline phosphatase (AP) at days 20, 30, and 40 after nucleofection with the reprogramming factors. Human primary colonies appeared earlier and at much higher numbers than rhesus primary colonies (Figure 3B,C).

The difference was particularly evident at day 20. The number of human AP-positive colonies was 10–50 times higher than the number of rhesus colonies (Figure 3C). At the following time points, the differences in the numbers became smaller due to the delayed appearance of more rhesus cell colonies and the fusion of the expanding colonies of the human cells, which led to a reduced number of discernable colonies.

In this study, we generated and characterized in total five rhesus macaque iPSC lines (from four adults (age range 5.7 to 8.4 years) and one neonatal), one from an adult female baboon and two from adult human donors (one male, one female) as a reference. PCR experiments using four pairs of primers, which are complementary to four different regions present in all three plasmids (Figure 3A) demonstrated the absence of plasmid DNA in 7 out of 8 generated iPSC lines between passage 15 and 20 (Figure 3D). Only one rhesus iPSC line (DPZ_iRh33.1) showed PCR signals even at higher passages suggesting genomics integration of a plasmid.

In summary, using our approach, fibroblasts from adult rhesus macaque were less efficiently reprogrammable than adult human fibroblasts under identical conditions. However, integration- and feeder-free reprogramming of rhesus fibroblasts was successful and reproducible. Using this approach, we reprogrammed rhesus macaque, baboon, and human fibroblasts.

### 3.2. Long-Term Culture of NHP-PSCs

**Figure 4:**
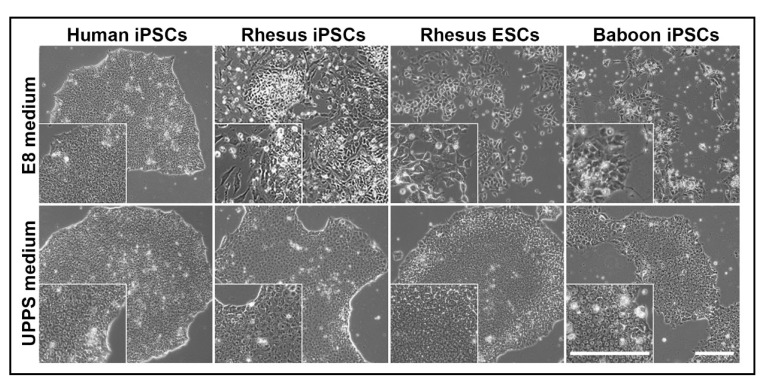
Morphology of human, rhesus, and baboon PSCs cultured feeder-free in Essential 8 (E8) medium and Universal Primate Pluripotent Stem Cell (UPPS) medium. Human iPSCs grow as compact colonies showing the typical primed PSC morphology when cultivated in both, E8 and UPPS medium. In contrast, baboon and rhesus iPSCs differentiate spontaneously when cultured in E8 medium but maintain undifferentiated compact colonies in UPPS medium. Adaptation of rhesus ESCs (line R344.6) from feeder-dependent to feeder-free condition failed in E8 medium. Cultured in UPPS medium, rhesus ESCs form compact undifferentiated colonies. Scale bars: 200 μm.

The newly generated rhesus and baboon iPSCs were initially (at lower passage numbers) cultivated in commercially available media developed for the culture of human PSCs, namely Essential 8 (E8) medium. Human iPSC lines were generated and cultured in parallel as a reference. In addition, we included the rhesus monkey embryonic stem cell (ESC) line 366.4 [45]. The E8 medium allowed long-term culture of undifferentiated human iPSCs (Figure 4).

However, the NHP-iPSCs showed signs of differentiation when cultured in E8 medium after a few passages (Figure 4, upper panel). Also, the adaptation of rhesus ESCs originally cultured on feeder cells to feeder-free conditions using E8 medium failed. A loss of the typical compact colony formation was observed soon, particularly when compared with undifferentiated human iPSCs under the same conditions (Figure 4, upper panel). The morphology of individual NHP cells changed, exhibiting a lower nucleus-to-cytoplasm ratio when compared with the human iPSCs (Figure 4, upper panel, insets), indicating progressing spontaneous differentiation. We then tested a second human PSC culture medium, named StemMACS iPS Brew XF, leading to similar results (data not shown for iPS Brew XF). Altogether, morphological differences between the human iPSCs and the NHP-PSCs in identical commercial media under feeder-free conditions were evident, although their morphology was reported to be similar under conventional feeder-based culture conditions [45].

Based on these findings, we systematically tested the supplementation of these two basal media with different combinations of small molecules and BMP4 to develop a medium that allows the undifferentiated feeder-free expansion of both, NHP- and human PSCs (Table 4).

Eventually, the supplementation of StemMACS iPS Brew XF medium with the Wnt signaling inhibitor IWR-1 and the GSK3 inhibitor CHIR99021 allowed the undifferentiated long-term cultivation of NHP-PSCs even in the absence of feeder cells (condition #8, Table 4). We then stepwise decreased the concentrations of IWR-1 (from initially 2.5 to 1 µM) and CHIR99021 (from initially 3 to 0.5 µM, condition #9, Table 4) in order to determine the minimum concentration needed to keep the NHP-PSCs in an undifferentiated state for more than 50 passages. We named this CHIR99021/IWR-1-supplemented StemMACS iPS Brew XF medium Universal Primate Pluripotent Stem Cell (UPPS) medium. The generated rhesus and baboon iPSCs, as well as the successfully adapted rhesus ESCs, could be cultivated in the UPPS medium without showing morphological signs of differentiation (Figure 4, lower panel). Human iPSCs were cultivated in this medium as a control.

### 3.3. NHP-iPSCs Cultured in UPPS Medium Are Pluripotent

**Figure 5:**
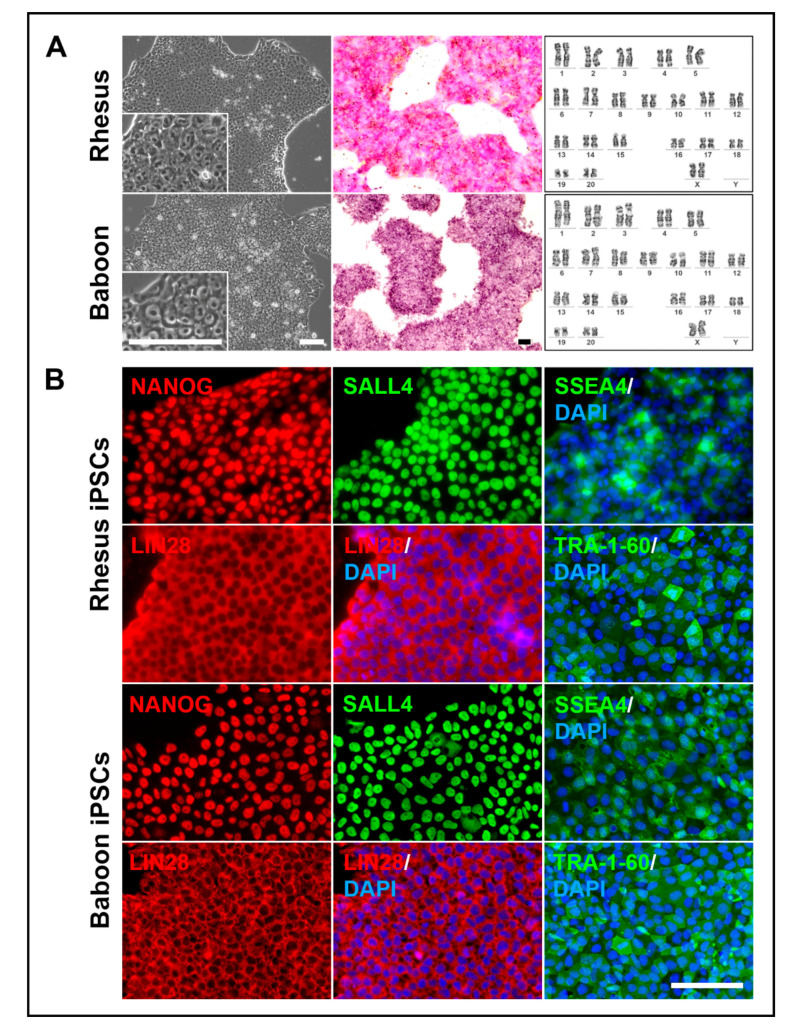
Characterization of transgene-free NHP-iPSCs. (**A**) Rhesus and baboon iPSCs cultured under feeder-free conditions (left) demonstrate alkaline phosphatase activity (middle) and show a normal karyotype (right). (**B**) Rhesus (upper panel) and baboon (lower panel) iPSCs express pluripotency related transcription factors NANOG and SALL4, pluripotency related surface markers SSEA4 and TRA-1-60 as well as LIN28. Scale bars: 100 μm.

The generated NHP-iPSCs were cultured in our developed UPPS medium on geltrex-coated dishes, i.e., these cells were at no time co-cultivated with xenogenic feeder cells. Even after long-term culture (over 50 passages) in UPPS medium the rhesus, baboon, and human (as a reference) iPSCs still form well-defined colonies (Figure 4, lower panel, Figure 5A, left) and show similar, primed PSC-specific morphologies with a high nucleus-to-cytoplasm ratio and distinct nucleoli (Figure 5A, left, insets).

The cells express alkaline phosphatase (Figure 5A, middle) and show a normal karyotype even after long-term cultivation of more than 30 passages (Figure 5A, right). Only one female rhesus macaque line (DPZ_iRh25.4) displayed a mosaic of X chromosome di-/trisomy, while fibroblasts of this animal, isolated from three independent sites (back skin, ventral skin, skin from the leg), were diploid. Immunofluorescence staining of the NHP-iPSCs showed stable expression of the pluripotency factors NANOG and SALL4 (which are not encoded by the reprogramming plasmids), the cytoplasmic protein LIN28 as well as the surface markers SSEA4 and TRA-1-60 (Figure 5B).

Reverse transcription PCR analyses of two selected rhesus iPSC lines (DPZ_iRh33.1 and DPZ_iRh34.1) show the switch from exogenous to endogenous pluripotency-related gene expression at selected time points during and after the reprogramming process (Figure S1). Exogenous gene expression of plasmid encoded *OCT4*, *KLF4* and *LIN28* was detectable in fibroblasts two days after nucleofection, while endogenous expression of *NANOG*, *OCT4*, *SOX2* and *LIN28* was not yet detectable at that time point (Figure S1). After passage 15, both cell lines had lost exogenous gene expression. The endogenous expression of the pluripotency factors *NANOG*, *OCT4*, *SOX2,* and *LIN28* was observed in both cell lines in all three passages tested. The established rhesus ESC line 366.4 [45] was used as positive control.

**Figure 6:**
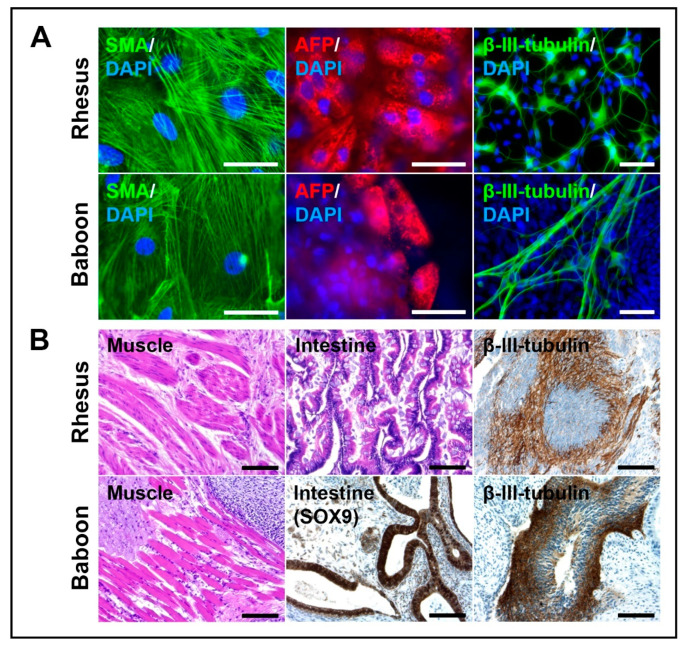
Differentiation potential of NHP-iPSCs. (**A**) Immunostaining of spontaneously differentiated rhesus (upper panel) and baboon (lower panel) iPSCs reveal smooth muscle actin (SMA, left, marker for mesoderm), α-feto protein (AFP, middle, marker for endoderm) and β-III-tubulin (right, marker for ectoderm) expressing cells, representing the three germ layers. Scale bars: 50 μm. (**B**) Teratoma formation of rhesus (upper panel) and baboon (lower panel) iPSCs after injection into immunodeficient mice, contain derivatives of mesoderm (muscle tissue, left), endoderm (intestinal tissue, middle, for baboon stained with SOX9, a primitive endodermal epithelial marker), and ectoderm (neural tissue, stained with β-III-tubulin). Scale bars: 100 μm.

To test whether the NHP-iPSCs are truly pluripotent, we differentiated them in vitro using the embryoid body formation method. Plated bodies from rhesus and baboon iPSCs differentiated spontaneously into smooth muscle actin, α-fetoprotein and β-III-tubulin expressing cells, representing the three germ layers (Figure 6A).

In vivo differentiation by teratoma formation corroborated the in vitro differentiation assays. Teratomas contained, beside others, muscle, intestinal epithelial and neural tissues, representing mesoderm, endoderm, and ectoderm, respectively (Figure 6B). Immunostaining against neuronal-specific β-III-tubulin and SOX9 (primitive endodermal epithelium; Figure 6B) verified ectodermal and endodermal tissue, respectively. These data demonstrate that the NHP-iPSCs cultured under chemically defined UPPS medium conditions are pluripotent.

### 3.4. NHP-iPSC-Derived Cardiomyocyte Characterization

**Figure 7:**
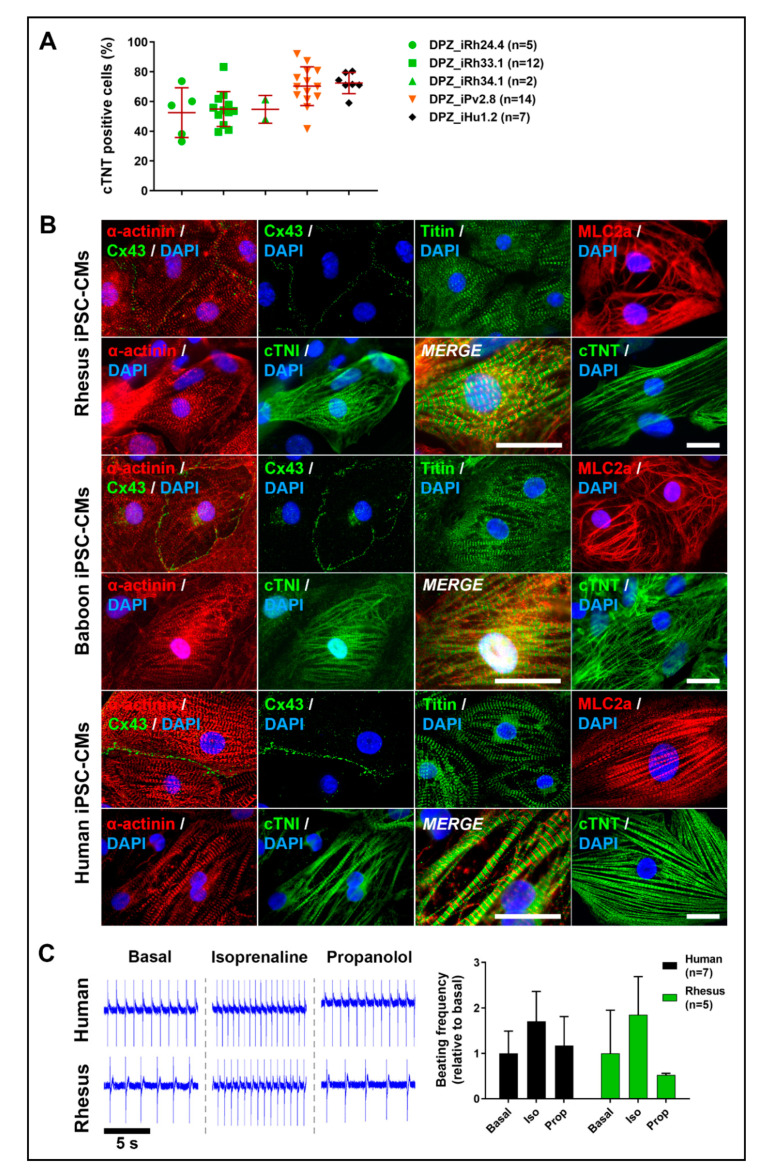
Directed cardiac differentiation of human and NHP-iPSCs. (**A**) Differentiation efficiencies of rhesus, baboon, and human iPSCs reflected by flow cytometric cTNT measurements at day 12 before metabolic selection. (**B**) Immunofluorescence staining of cardiac-specific proteins show structure and morphology of rhesus and baboon iPSC-derived cardiomyocytes: sarcomeric α-actinin, cardiac troponin I (cTNI), cardiac troponin T (cTNT), connexin 43 (Cx43), myosin light chain a (MLC2a) and titin. Scale bars: 20 μm. (**C**) Rhesus and human iPSC-derived cardiomyocytes respond to isoprenaline (increased beating frequencies compared to basal) and propanolol (decreased beating frequencies compared to isoprenaline treatment). Beating frequencies were analyzed by measuring the field potentials with the microelectrode array (MEA) system. Excerpts of original recordings are shown on the left. The graph shows fold change of beating frequencies when treated with isoprenaline and propranolol relative to basal recordings.

Several directed 2D monolayer cardiac differentiation protocols have been established for human PSCs [41,46,47,48,49,50]. We first tried to adopt the small molecule-based differentiation protocols lacking growth factors to the rhesus and baboon iPSCs. We tested CHIR99021 and Wnt agonists IWR-1 or IWP-2 in different concentrations and timings in different media (full list of conditions tested see Table 2). However, only sporadically, the NHP-iPSCs developed very low cardiomyocyte content. In contrast, the human iPSC reference lines efficiently and robustly differentiated into cardiomyocytes. We then combined the small molecule protocol with the growth factors BMP4 and activin A (Figure 2). With this hybrid method, we successfully and robustly differentiated NHP-iPSCs into cardiomyocytes. First beating cardiomyocytes from rhesus, baboon, and human cells could be observed at day 7 or 8 of the differentiation protocol (Videos S1 and S2). Flow cytometric analyses of cTNT positive cells before metabolic selection revealed average cardiac differentiation efficiencies between 53% and 72% at day 12 of differentiation (rhesus (53%), baboon (70%), human (72%); Figure 7A).

The morphology, size, structure as well as cardiac-specific gene expression of 2-month-old rhesus and baboon iPSC-derived cardiomyocytes were similar and showed the typical immature, embryonic-like morphology reported for human PSC-derived cardiomyocytes (Figure 7B). Immunofluorescence staining of sarcomeric α-actinin, cardiac troponin T, cardiac troponin I and titin reveal the immature, relatively unorganized striated pattern of sarcomere structures (in contrast to adult cardiomyocytes with a parallel-organized pattern). The NHP cardiomyocytes express the gap junction protein connexin 43, which is important for the electrical cell-to-cell coupling. We used the microelectrode array (MEA) system to functionally characterize rhesus compared to human iPSC-derived cardiomyocytes (Figure 7C). Spontaneous field potentials were recorded before and after treating the cells with the non-selective β-adrenoreceptor agonist isoprenaline (100 nM). Isoprenaline caused the anticipated increase in the beating rate of the cardiomyocytes relative to basal (untreated) conditions (in average 1.7- and 1.8-fold change in the beating frequencies for human (n = 7) and rhesus (n = 5) iPSC-CM, respectively; Figure 7C). After five minutes of treatment with isoprenaline, the non-selective β-adrenoreceptor antagonist propanolol (2 µM) was added to the medium and reversed the chronotropic effect of isoprenaline back to near basal conditions in both, rhesus and human iPSC-derived cardiomyocytes.

**Figure 8:**
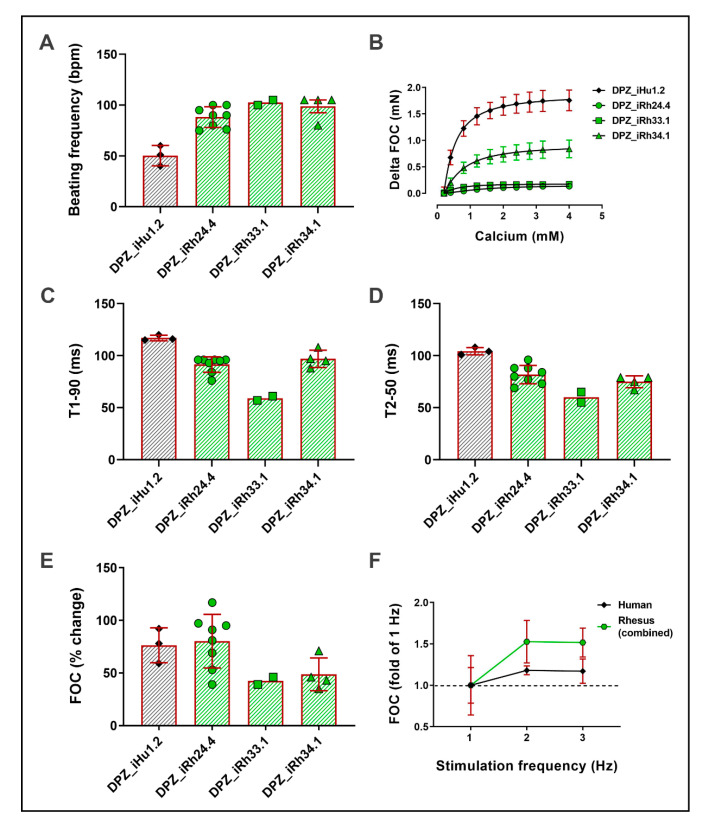
Functional characterization of primate engineered heart muscle (EHM). (**A**) EHM beating frequency (bpm) after four weeks of EHM culture. (**B**) Force of contraction (FOC) of EHM in response to increasing extracellular calcium concentrations. Displayed as delta of increase from 0.2 mM calcium. (**C**) The time to 90% contraction of the EHM reversely correlates with the beating frequency. (**D**) The time to 50% relaxation correlates with the 90% contraction time. (**E**) Primate EHM inotropic response to acute isoprenaline (1 µM) stimulation given as % change in the force of contraction. (**F**) Force frequency response of human and rhesus EHM (combined data of the four rhesus lines).

To validate the contractility of the iPSC-derived cardiomyocytes, we generated rhesus and human engineered heart muscle (EHM). Highly enriched rhesus cardiomyocyte populations (94% ± 1% sarcomeric α-actinin-positive, n = 4) were obtained by metabolic selection comparable to human cardiomyocytes (89% sarcomeric α-actinin-positive, n = 1). In line with in vivo observations, spontaneous beating frequency was significantly higher in rhesus EHM (DPZ_iRh24.4: 88 ± 4 bpm; DPZ_iRh33.1: 103 bpm; DPZ_iRh34.1: 99 ± 6 bpm) than in human EHM, which showed spontaneous beating at 50 ± 10 bpm (Figure 8A).

All rhesus EHM displayed a positive inotropic response to extracellular calcium comparable to the human EHM suggesting a similarly developed electromechanical coupling. Absolute contractile force was, however, variable depending on the rhesus iPSC line, and significantly lower than in human EHM (Figure 8B). As to contraction kinetics and in line with the higher beating rate, contraction and relaxation were accelerated in rhesus EHM compared to human EHM (Figure 8C,D). Catecholamine stimulation with 1 µM isoprenaline induced a clear positive inotropic response in rhesus EHM from all lines (Figure 8E). In summary, NHP-iPSC-derived cardiomyocytes exhibit species-specific contractile properties (higher beating rate compared to human) and respond to inotropic interventions such as calcium and isoprenaline in a similar way as human EHM.

## 4. Discussion

Pluripotent stem cells, including iPSCs, revolutionize different fields of biomedicine, including potential treatments of so far incurable diseases. In fact, several late preclinical animal studies involving NHP have been conducted [17,19,21,23,38], and the first PSC-based ATMPs are under clinical investigation, for example, for the treatment of age-related macular degeneration [51], Parkinson’s disease [52], and heart disease [53]. There are expectations that, in the future, PSCs might be used routinely as a therapeutic agent in the clinic. However, therapies are currently still experimental, and many open questions remain. Large animal models are needed in which the relevant questions regarding the clinical treatment path, including immunological considerations pertaining to auto- and allografting, can be adequately addressed. As to this end, NHP appear to date the only relevant model because NHP-ESCs and iPSCs can be robustly derived and tested in the homologous model system. Other advantages of NHP as a late preclinical model include their close phylogenetic relationship to humans, which is reflected by comparable anatomy, immune systems, and genetic and transcriptomic constitution as well as life span [2,3,4,6]. From a clinical perspective, allogeneic and autologous transplantations, using NHP as model organisms, may also provide a deeper understanding of the respective immunological responses and the corresponding immunosuppression regimens for PSC-derived transplants in humans (for review see [54]). In the present study, we demonstrate the derivation of novel rhesus and baboon iPSC lines using integration- and feeder-free conditions. Most importantly, rhesus and baboon iPSCs could be maintained in an undifferentiated state for extended passages and directed to differentiate into cardiomyocytes to demonstrate one potential use.

We reprogrammed fibroblasts from five rhesus macaques, one baboon, and two humans to demonstrate the broad applicability of our reprogramming method. To our knowledge, most of the relatively few NHP-iPSC lines generated until now (including macaque, baboon, gorilla, chimpanzee, bonobo, and common marmoset) were typically reprogrammed using methods involving genomic integration of the reprogramming vectors [23,29,30,33,34,55,56,57,58,59]; or genomic integration followed by the excision of the lentiviral vector leaving a genetic tag in the host genome [26,60]. So far, to our knowledge, only very few studies using an approach based on non-integrating vectors were reported recently [16,21,24,27]. Human iPSCs with genomic integration of the reprogramming vectors are non-preferred for therapeutic applications in patients. Regarding the translational value of NHP, we decided to generate genomically non-modified primate iPSCs using episomal plasmid vectors. The episomal system involves p53 suppression and non-transforming L-MYC instead of c-MYC [40]. Only in one out of eight generated iPSC lines (DPZ_iRh33.1), the reprogramming vector DNA was detected, which is in line with the original publication where genomic integration of the vector DNA was reported in two out of seven human iPSC lines [40]. However, even though the majority of the cell lines in the present study was transgene-free, it is still important to karyotype the cells after extended periods of culture to confirm the long-term genomic stability.

An interesting observation of our study was that the NHP cells were less efficient in reprogramming compared to the human cells. The reason for this is currently unknown. One possible explanation could be that the human reprogramming factors may work less efficiently in the NHP cells than in the human cells. However, the reprogramming factors are highly conserved between humans and rhesus macaques with identities on the protein level of 99% (OCT4, SOX2, KLF4, LIN28) and 96% (L-MYC) (Table S1). Furthermore, the human p53 shRNA sequence is 100% complementary to the respective rhesus sequence. In a previous study, we used marmoset open reading frames cloned into a stably expressing piggyBac transposon to reprogram neonatal marmoset fibroblasts and obtained also only very few primary colonies [33]. Recently, we also generated baboon iPSCs using the same piggyBac vector and again only a rather small number of primary colonies was observed [44]. Finally, it has been shown that mouse factors are sufficient to efficiently reprogram human cells and vice versa [61,62]. Taken together, all this strongly argues against the hypothesis that the human reprogramming factors are responsible for the low reprogramming efficiencies of the rhesus (and the baboon) cells compared to the human cells. Further investigations are necessary to draw final conclusions. 

We reprogrammed NHP cells using the same method as for human cells with a chemically defined culture medium. While previous studies used xenogenic feeder cells for the generation and cultivation of NHP-iPSCs [16,27,28,29,33,34,56,58,60,63,64], we succeeded, to our knowledge, for the first time in the generation of NHP-iPSCs without the support of xenogenic feeder cells. The generation of human and NHP-iPSCs was successful in our study using the well-defined Essential 8 culture medium developed for human stem cell culture [65]. As expected, the generated human iPSCs could be cultured in this medium while keeping their pluripotent state. However, the quantitatively undifferentiated long-term cultivation of the NHP-iPSCs was not possible in our hands due to continuous spontaneous differentiation of many colonies, which made it necessary to manually pick the few remaining undifferentiated iPSCs. To exclude that our NHP-iPSCs were incompletely reprogrammed, we tried to culture the rhesus ESC line R366.4 [45] in Essential 8 medium. However, the rhesus ESC line also differentiated spontaneously when cultured feeder-free in Essential 8 medium. The same finding was made using StemMACS iPS Brew XF. This showed that the two commercially available media are not sufficient to keep rhesus and baboon PSCs in a stable undifferentiated pluripotent state. We then tested amongst other factors the supplementation of both culture media with Wnt response-1 (IWR-1), a Wnt signaling inhibitor. It was shown previously that IWR-1 could optimize the derivation and long-term cultivation of mouse epiblast stem cells (EpiSCs) in combination with FGF2 in N2B27 media [66]. Mouse EpiSCs are considered as the in vitro counterparts of human (primed) ESCs. Wu et al. postulated the supportive effect of IWR-1 also on human ESCs, rhesus PSCs and chimpanzee iPSCs. The combined addition of IWR-1 and BMP4 to Essential 8 and StemMACS iPS-Brew XF media was tested as a second condition. It was shown that BMP4 is secreted by mouse embryonic fibroblasts and was identified as a pluripotency- and self-renewal promoting factor for mouse ESCs [67]. As a third condition, we tested IWR-1 combined with the GSK-3 inhibitor (and hence Wnt pathway activator) CHIR99021. Kim and colleagues could show that the dual administration of CHIR99021 and Wnt inhibitor XAV939 or CHIR99021 and IWR-1 allowed long-term maintenance of undifferentiated mouse EpiSCs as well as human ESCs. These cells were routinely maintained on mouse embryonic fibroblasts in semi-defined cell culture media using Knockout serum replacement [68]. Of all tested conditions, the combination of CHIR99021 and IWR-1 in StemMACS iPS Brew XF medium turned out to be the only condition in our present study facilitating consistent undifferentiated long-term self-renewal (>50 passages) of rhesus, baboon, and human iPSCs. Eventually, we decreased the concentrations from 3 µM CHIR99021 and 2.5 µM IWR-1, as originally published by Kim and colleagues, to 0.5 µM CHIR99021 and 1 µM IWR-1. We aimed for a reproducible and robust primate iPSC generation and cultivation protocol that allows direct experimental comparisons of human and NHP-PSCs under identical starting conditions making biological effects and cellular differences between the species probably more apparent. To our knowledge, we report for the first time the generation and long-term cultivation of rhesus and baboon iPSCs under feeder-free conditions using a well-defined and serum-free cultivation medium. Navara and colleagues recently optimized the culture condition for baboon PSCs, but this culture system was still dependent on the co-cultivation with mouse embryonic fibroblasts as feeder cells [28]. Also, Zhang and colleagues could recently show the integration-free generation of rhesus iPSCs using the episomal plasmid vector approach [64]. However, these rhesus iPSCs were generated and initially maintained on feeder cells with a semi-defined cultivation medium using Knockout serum replacement. Interestingly, the attempt reported by Zhang and colleagues to culture the rhesus iPSCs feeder-free in the semi-defined culture medium supplemented with CHIR99021 and IWR-1 failed to keep the cells in an undifferentiated pluripotent state. Only the combination with CHIR99021, IWR-1 and activin A supported long-term self-renewal of the rhesus iPSCs. Our fully defined supplemented StemMACS iPS Brew XF medium does not include activin A but supports rhesus, baboon, and human PSCs in maintaining their pluripotency during long-term cultivation.

PSCs play an important role in (comparative) developmental biology as well as for regenerative medicine. For these purposes, it is crucial to establish efficient protocols for the directed differentiation of NHP-PSCs into specific cell types. Here, we tested different protocols for cardiac differentiation, originally established for human PSCs. As for the cultivation of human and NHP-PSCs, we also aimed for a universal cardiac differentiation protocol for better comparability between the primate species. We tested several growth factor-free cardiac differentiation protocols using a small molecule-based induction of differentiation, for example, according to [46]. However, in contrast to the growth factor-free cardiac differentiation protocols that work for human PSCs, the combination of Wnt signaling activation together with BMP4 and activin A treatment followed by Wnt signaling inhibition was required to achieve efficient and reproducible cardiac differentiation in our NHP-iPSCs. Finally, we came up with a NHP cardiomyocyte differentiation protocol slightly modified compared to the protocol developed previously by us for human cells [41]. First beating NHP-iPSC-derived cardiomyocytes could be observed around day eight of differentiation. Flow cytometry analyses at day 12 before metabolic selection revealed higher differentiation efficiencies in baboon (70%) and human (72%) compared to rhesus (53%) cells. So far, we did not perform a systematic analysis of the non-cardiomyocyte cells at this stage of differentiation. However, initial RT-PCR analyses may suggest the presence of cardiac mesodermal progenitors (based on the presence of *GATA4*, *PDGFRα* and *PDGFRβ*), cardiac fibroblasts (*VIM*, *CD90* [*THY1*], *POSTN*, *DDR2, COL3A1, COL1A1,* and *COL1A2* with *COL3A1* and *COL1A2* as the main collagen subtypes in the heart extracellular matrix) and the development of endothelial or smooth muscle cells (*PECAM1* [*CD31*] and *CD34*) (data not shown). To reduce the heterogeneity of the cell population for down-stream analyses and applications we applied metabolic selection resulting in cultures highly enriched in cardiomyocytes. The fact that Wnt modulation using CHIR99021 alone is not sufficient to differentiate the NHP-iPSCs shows a clear difference of rhesus and baboon PSCs compared to human PSCs. There are only very few publications available showing directed cardiac differentiation protocols for NHP-PSCs to compare with our findings. Shiba and colleagues used the so-called matrix sandwich method, which also involves BMP4 and activin A treatment, to differentiate cynomolgus macaque iPSC line into cardiomyocytes [21]. However, the average differentiation efficiencies were relatively low (20% cTNT positive cells before metabolic selection), and only one iPSC line was used in this study. Zhao and colleagues reported also only one rhesus iPSC line but did not mention the differentiation efficiency using their protocol [24]. Considering iPSC line heterogeneity, we posit that side by side studies of NHP-iPSC lines are valuable to establish generally valid and robust protocols for directed differentiation of NHP-PSCs.

Preliminary observations indicate that the generated cardiomyocytes have typical features of fetal-like cardiomyocytes. Most of the cells are mono-nucleated, have a network-like sarcomere structure and a roundish shape (see Figure 7B). Additionally, we comparatively estimated the relative transcript abundance of selected cardiomyocyte maturation markers by RT-PCR in iPSC-CM and native rhesus heart. These preliminary data (e.g., *RyR2* and *KCND3; data not shown)* support the view that the iPSC-CM are not mature. A deeper analysis of the maturation stage of the generated cardiomyocytes, ideally by single cell transcriptome analyses, is required to complete the characterization of the NHP-iPSC-CMs.

To ensure the functionality of the cardiomyocytes derived from UPPS-cultured iPSCs, we performed MEA analysis and generated EHMs [41]. Both assays show the functionality of the β-adrenoreceptor signaling pathway. Whilst the MEA experiments could identify classical chronotropic responses, mediated via HCN4-expressing nodal-like cells, the EHM study provided evidence for a direct effect on cardiomyocyte contractility (positive inotropic effects). The latter are rarely observed in the literature suggesting that in 2D culture mostly nodal cells or embryonic cardiomyocytes with detectable pacemaker (funny) current are generated, whilst in EHM maturation working myocardium is achieved. The chronotropic response in EHM is likely mediated via a minor nodal cell component that functions as a surrogate pacemaker for the mostly ventricular-like cardiomyocytes with a stable phase four of the action potential. 

Finally, we conclude that feeder-free maintenance of NHP-PSC is supported by GiWi as provided in our UPPS protocol. Directed differentiation can most likely be further improved by a modification of the growth factor/GiWi protocol. The established rhesus and baboon lines will be available for use for in vitro and in vivo studies such as the simulation of clinical heart muscle repair in a homologous NHP model.

## Figures and Tables

**Table 1:** Primary and secondary antibodies used in this study.

Name	Vendor	Catalogue	Dilution
α-feto protein	Dako	A0008	1:100
α-actinin	Sigma-Aldrich	A7811	1:1000
β-tubulin III	Sigma-Aldrich	T8660	1:1000
Connexin 43 (Cx43)	Abcam	ab11370	1:1000
Cardiac troponin I (cTNI)	Abcam	ab47003	1:200
Cardiac troponin T (cTNT) ^1^	Miltenyi Biotec	130-106-687	1:10
Cardiac troponin T (cTNT) ^2^	Thermo Fisher	MS295PABX	1:200
LIN28	R&D Systems	AF3757	1:300
Myosin light chain 2a (MLC2a)	Synaptic Systems	311-011	1:200
NANOG	Cell Signalling	4903	1:400
SALL4	Abcam	ab57577	1:200
Smooth muscle actin	Sigma-Aldrich	A2547	1:1000
SOX9	Merck	AB5535	1:1000
SSEA4	Abcam	ab16287	1:200
Titin	Merck	MAB1553	1:50
TRA-1-60	Abcam	ab16288	1:200
Alexa555-goat-α-mouse IgG	Thermo Fisher	A21424	1:1000
Alexa488-goat-α-mouse IgG	Thermo Fisher	A11029	1:1000
Alexa488-goat-α-mouse IgG/IgM	Thermo Fisher	A10680	1:1000
Alexa488-donkey-α-goat IgG	Thermo Fisher	A11055	1:1000
Alexa488-donkey-α-rabbit IgG	Thermo Fisher	A21206	1:1000

^1^ Used for flow cytometry. ^2^ Used for immunocytochemistry.

**Table 2:** Tested conditions for directed cardiac differentiation of NHP-PSCs.

Cultivation Media	Mesodermal Induction	Cardiac Induction	Reference
Day 0–5: RPMI 1640 Day ≥ 6: RPMI 1640 + B27	1 µM CHIR99021 (48 h) or 2.5 µM CHIR99021 (48 h) or 4 µM CHIR99021 (48 h) or 5 µM CHIR99021 (48 h)	5 μM IWP-2 (48 h) or 5 μM IWR-1 (48 h)	Modified after Lian et al., 2015
Day 0–7: RPMI 1640 + L-ascorbic acid 2-phosphate (690 µM) + recombinant human albumin (0.5 mg/mL) Day ≥ 8: RPMI 1640 + B27	1 µM CHIR99021 (48 h) or 2.5 µM CHIR99021 (48 h) or 4 µM CHIR99021 (48 h) or 5 µM CHIR99021 (48 h)	5 μM IWP-2 (48 h) or 5 μM IWR-1 (48 h)	Modified after Burridge et al., 2014
Day 0–6: RPMI 1640 + B27 (minus insulin) Day ≥ 7: RPMI 1640 + B27	1 µM CHIR99021 (24 h) or 3 µM CHIR99021 (24 h) or 4 µM CHIR99021 (24 h) or 8 µM CHIR99021 (24 h) or9 µM CHIR99021 (24 h) or 10 µM CHIR99021 (24 h)	5 μM IWP-2 (48 h) or 5 μM IWR-1 (48 h)	Modified after Lian et al., 2013
Day 0–6: IMDM + B27 (minus insulin) Day ≥ 7: RPMI 1640 + B27	1 µM CHIR99021 (24 h) or 3 µM CHIR99021 (24 h) or 4 µM CHIR99021 (24 h) or 8 µM CHIR99021 (24 h) or 9 µM CHIR99021 (24 h) or 10 µM CHIR99021 (24 h)	5 μM IWP-2 (48 h) or 5 μM IWR-1 (48 h)	Modified after Lian et al., 2013
Day 0–6: RPMI 1640 + B27 (minus insulin) + sodium pyruvate (1 mM) + L-ascorbic acid 2-phosphate (200 µM) Day ≥ 7: RPMI 1640 + B27 + L-ascorbic acid 2-phosphate (200 µM)	1 µM CHIR99021 + 9 ng/mL activin A + 5 ng/mL BMP4 (36 h)	5 μM IWR-1 (96 h)	Modified after Tiburcy et al., 2017

**Table 3:** Oligonucleotides used in this study.

Name	Sequence	F (bp) ^1^	T (°C) ^2^	C ^3^
*WPRE* (1)	for: 5′-GCT ATT GCT TCC CGT ATG GC-3′ rev: 5′-CAA AGG GAG ATC CGA CTC GT-3′	470	54	32
*EBNA-LoxP* (2)	for: 5′-AAG AGG AGG GGT CCC GAG A-3′ rev: 5′-GCC AAT GCA ACT TGG ACG TT-3′	555	61	32
*OriP1* (3)	for: 5′-GGT TCA CTA CCC TCG TGG AAT-3′ rev: 5′-CGG GGC AGT GCA TGT AAT-3′	592	57	32
*OriP2* (4)	for: 5′-GGT GAC TGT GTG CAG CTT TG-3′ rev: 5′-GGA GCT GAG TGA CGT GAC AA-3′	416	54	32
*β-actin* ^4^	for: 5′-GAC CTG ACT GAC TAC CTC ATG-3′ rev: 5′-GGT AGT TTC GTG GAT GCC ACA-3′	379/380	61	32
*KLF4* (exo)	for: 5′-TTC ATC GAC GAG GCT AAG CG-3′ rev: 5′-TCA CTG ACA GCC ATG GTG AA-3′	812	53	30
*OCT4* (exo)	for: 5′-TGA TCC TCG GAC CTG GCT AA-3′ rev: 5′-TCCCCGAAGCTTGAATTCGC-3′	1021	54	30
*OCT4* (endo)	for: 5′-GAG AAG GAG AAG CTG GAG CAA-3′ rev: 5′-ACA TCC TTC TCG AGC CCA A-3′	841	53	30
*LIN28* (exo)	for: 5′-ACT CAA ACT GGC TGG GGA TG-3′ rev: 5′-TTC AAG CTC CGG AAC CCT TC-3′	327	54	30
*LIN28* (endo)	for: 5′-GGG TGT TCT GTA TTG GGA GTG-3′ rev: 5′-GCA CCC TAT TCC CAC TTT CTC-3′	371	61	30
*NANOG*	for: 5′-CAG AGA TAC CTC AGC CTC CAG-3′ rev: 5′-CTT CAG GTT GCA TGT TCG T-3′	562	54	30
*SOX2* (endo)	for: 5′-GGT AGG AGC TTT GCA GGA AGT-3′ rev: 5′-CCA ACG ATG TCA ACC TGC ATG-3′	428	61	30
*β-actin* ^5^	for: 5′-TGG ATG ATG ATA TCG CCG CGC T-3′ rev: 5′-GGG CCT CGG TCA GCA GCA CGG-3′	324	61	20

^1^ Fragment length. ^2^ Annealing temperature. ^3^ Cycles. ^4^ Used in Figure 3. ^5^ Used in Figure S1.

**Table 4:** Tested conditions to develop a feeder-free cultivation method for both, NHP- and human PSCs.

Condition #	Culture Media	Supplements
1	Essential 8	-
2	Essential 8	2.5 µM IWR-1
3	Essential 8	2.5 µM IWR-1 25 ng/mL BMP4
4	Essential 8	2.5 µM IWR-1 3 µM CHIR99021
5	StemMACS iPS-Brew XF	-
6	StemMACS iPS-Brew XF	2.5 µM IWR-1
7	StemMACS iPS-Brew XF	2.5 µM IWR-1 25 ng/mL BMP4
8	StemMACS iPS-Brew XF	2.5 µM IWR-1 3 µM CHIR99021
9	StemMACS iPS-Brew XF	1 µM IWR-1 0.5 µM CHIR99021

## Supplementary Materials

The following are available online at https://www.mdpi.com/2073-4409/9/6/1349/s1, Figure S1: Switch from exogenous to endogenous pluripotency-related gene expression at selected time points during and after reprogramming, Table S1: Protein sequences of both human (h) and rhesus (r) pluripotency genes compared via SerialCloner Alignment. All values are given in percent of similarity, Video S1: Rhesus iPSC-derived cardiomyocytes before metabolic selection, Video S2: Baboon iPSC-derived cardiomyocytes before metabolic selection.

## Appendix A

Unsuccessful growth factor-free NHP cardiomyocyte differentiation protocols.

Approach 1: When reaching confluency of about 80–90%, the UPPS medium was replaced by mesodermal induction medium (Roswell Park Memorial Institute Medium (RPMI 1640, Thermo Fisher), 0.2 mg/mL L-ascorbic acid 2-phosphate (Sigma-Aldrich), 0.5 mg/mL recombinant human albumin (Sigma-Aldrich), with different concentrations of CHIR99021 (1, 2.5, 4, and 5 μM)). After 48 h, medium was replaced by cardiac induction medium (RPMI 1640, 0.2 mg/mL L-ascorbic acid 2-phosphate, 0.5 mg/mL recombinant human albumin, 5 μM IWP-2 (Millipore) or IWR-1). At day 4 and 6, medium was changed with RPMI 1640, 0.2 mg/mL L-ascorbic acid 2-phosphate, and 0.5 mg/mL recombinant human albumin. From day 8 on, medium was replaced by cardiomyocyte cultivation medium (RPMI 1640, B27 with insulin (Thermo Fisher)) and changed every 2–3 days. A similar protocol was tested with a slightly modified mesodermal induction medium using RPMI 1640 with Chir99021 only.

Approach 2: A second cardiac differentiation protocol used RPMI 1640 and B27 without insulin. At a confluence of about 80–90%, the UPPS medium was replaced by mesodermal induction medium (RPMI 1640, B27 without insulin (Thermo Fisher), with different concentrations of CHIR99021 tested (1, 3, 4, 8, 9 and 10 μM)). After 24 h, medium was changed with RPMI 1640, B27 without insulin. At day 3, medium was replaced by cardiac induction medium (RPMI 1640, B27 without insulin (half fresh, half left), 5 μM IWP-2 or IWR-1). At day 5, medium was changed with RPMI 1640, B27 without insulin. From day 7 on, medium was replaced by cardiomyocyte cultivation medium (RPMI 1640, B27 with insulin) and changed every 2-3 days. A similar protocol was tested using IMDM instead of RPMI 1640.
